# Causal factors underlying diabetes risk informed by Mendelian randomisation analysis: evidence, opportunities and challenges

**DOI:** 10.1007/s00125-023-05879-7

**Published:** 2023-02-14

**Authors:** Shuai Yuan, Jordi Merino, Susanna C. Larsson

**Affiliations:** 1 Unit of Cardiovascular and Nutritional Epidemiology, Institute of Environmental Medicine, Karolinska Institutet, Stockholm, Sweden; 2 Diabetes Unit and Center for Genomic Medicine, Massachusetts General Hospital, Boston, MA USA; 3 Programs in Metabolism and Medical and Population Genetics, Eli and Edythe L. Broad Institute of MIT and Harvard, Cambridge, MA USA; 4 Department of Medicine, Harvard Medical School, Boston, MA USA; 5 Novo Nordisk Foundation Center for Basic Metabolic Research, Faculty of Health and Medical Sciences, University of Copenhagen, Copenhagen, Denmark; 6 Unit of Medical Epidemiology, Department of Surgical Sciences, Uppsala University, Uppsala, Sweden

**Keywords:** Causality, Diabetes, Mendelian randomisation, Review, Risk factor

## Abstract

Diabetes and its complications cause a heavy disease burden globally. Identifying exposures, risk factors and molecular processes causally associated with the development of diabetes can provide important evidence bases for disease prevention and spur novel therapeutic strategies. Mendelian randomisation (MR), an epidemiological approach that uses genetic instruments to infer causal associations between an exposure and an outcome, can be leveraged to complement evidence from observational and clinical studies. This narrative review aims to summarise the evidence on potential causal risk factors for diabetes by integrating published MR studies on type 1 and 2 diabetes, and to reflect on future perspectives of MR studies on diabetes. Despite the genetic influence on type 1 diabetes, few MR studies have been conducted to identify causal exposures or molecular processes leading to increased disease risk. In type 2 diabetes, MR analyses support causal associations of somatic, mental and lifestyle factors with development of the disease. These studies have also identified biomarkers, some of them derived from the gut microbiota, and molecular processes leading to increased disease risk. These studies provide valuable data to better understand disease pathophysiology and explore potential therapeutic targets. Because genetic association studies have mostly been restricted to participants of European descent, multi-ancestry cohorts are needed to examine the role of different types of physical activity, dietary components, metabolites, protein biomarkers and gut microbiome in diabetes development.

**Graphical abstract:**

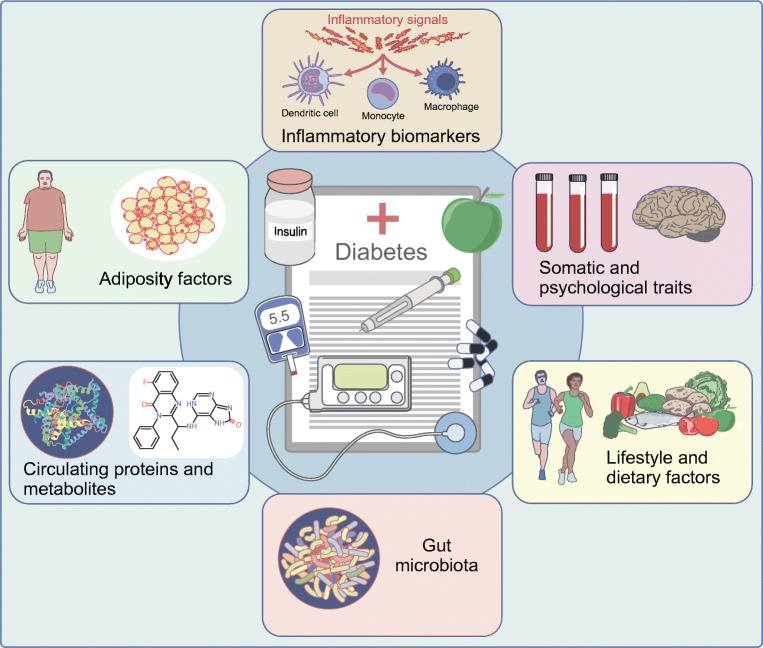

**Supplementary Information:**

The online version contains a slide of the figure for download available at 10.1007/s00125-023-05879-7.

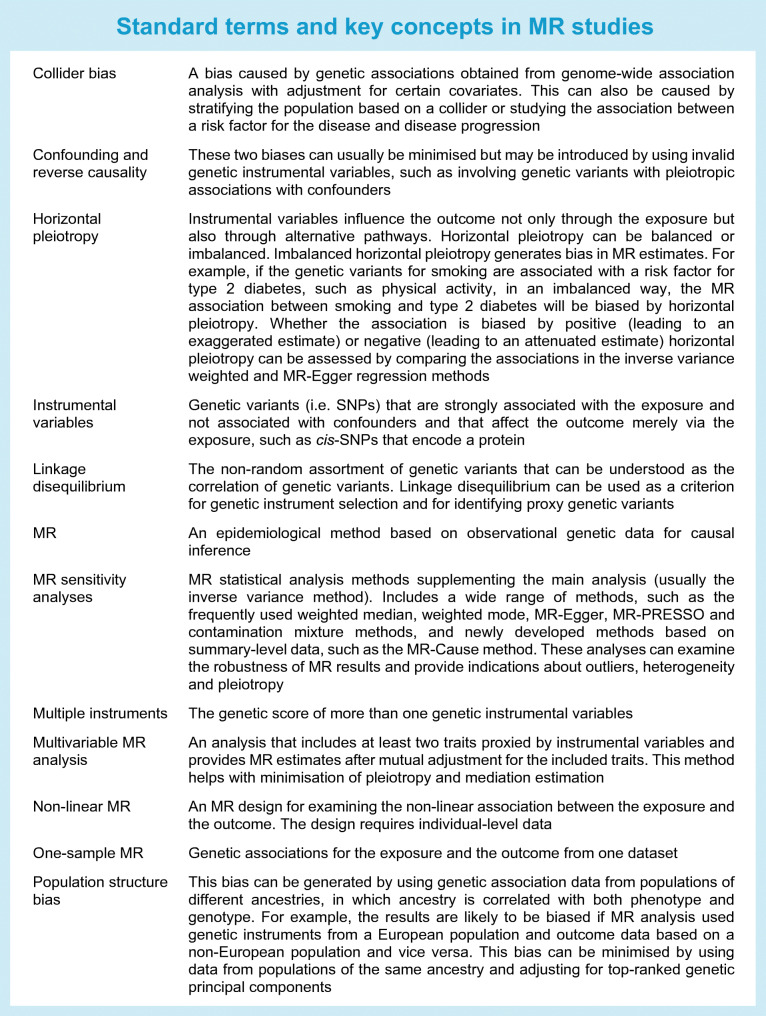

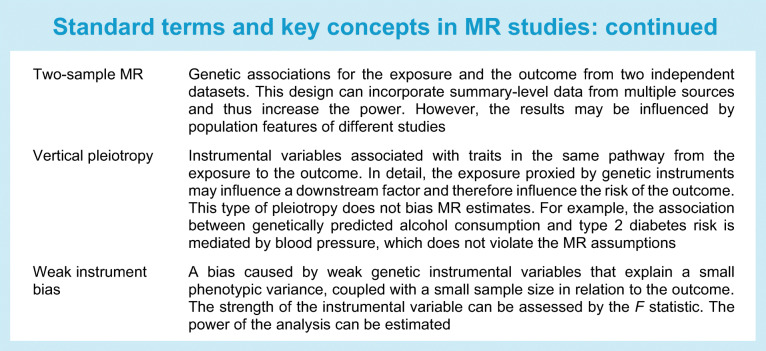





## Introduction

Diabetes is a leading health issue that causes severe disease and has a huge economic burden worldwide [1, 2]. Many epidemiological studies have assessed the causes of diabetes to provide an evidence base for disease prevention. For example, in type 2 diabetes, an exposure-wide umbrella review including 142 factors identified a wide range of biomarkers, medical conditions and dietary, lifestyle, environmental and psychosocial factors that were associated with the risk of disease [3]. The picture is somewhat different for type 1 diabetes owing to the strong genetic contribution and less influence of external factors. In addition to genetic factors, only a few environmental factors, including birthweight and childhood obesity, have been linked to type 1 diabetes [4]. While results from observational studies have provided initial evidence of potential exposures associated with diabetes, residual confounding and reverse causation limit our understanding of the complex set of factors underlying the development of diabetes. Thus, whether the factors observed in previous observational studies are causally associated with the risk of diabetes remains unconfirmed. A clear appraisal of the causal risk factors for diabetes is of great importance for disease prevention.

**Fig. 1:**
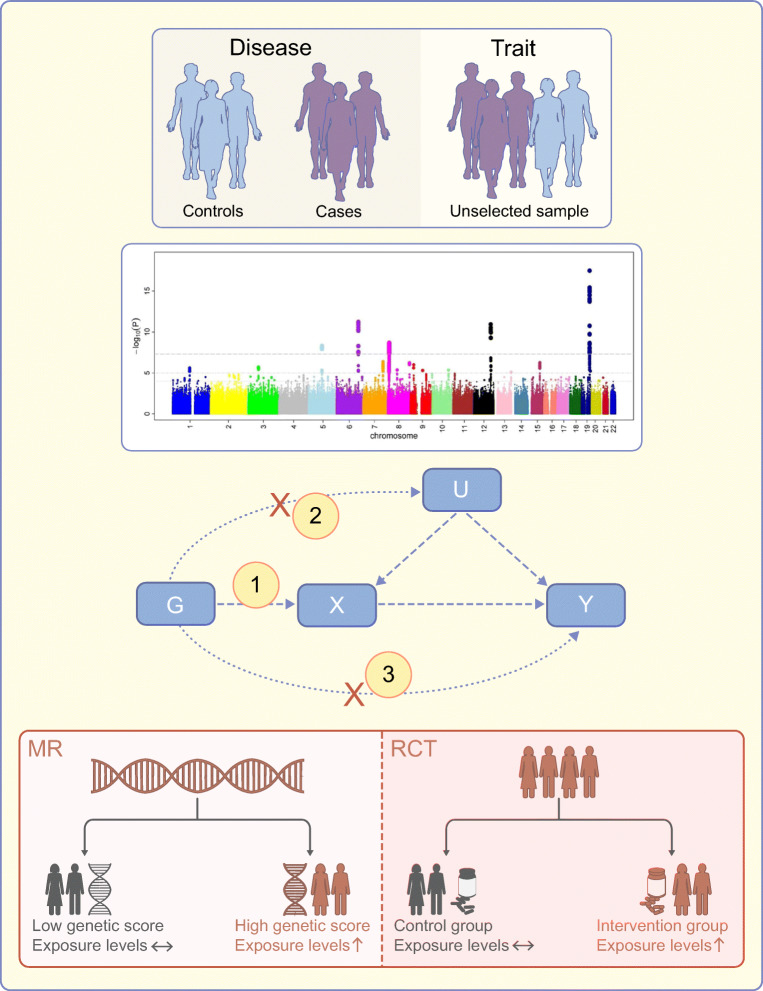
Study design and assumptions of MR analysis. The process of MR analysis is shown from top to bottom. In detail, MR analysis is based on genome-wide association analyses of the exposure and outcome. Genetic instruments for the exposure are independent SNPs that are strongly associated with the exposure of interest in a genome-wide association analysis in an unselected sample, such as a general population. Likewise, summary-level data on the outcome are obtained from a genome-wide association analysis of a binary phenotype that defines the population into cases and controls. The directed acyclic graph represents the study design and assumptions of MR analysis; G indicates the genetic instruments, X indicates the exposure of interest, Y indicates the outcome of interest, and U indicates the confounders. There are three important assumptions in MR analysis. Assumption 1 indicates that the genetic variants used as the instrumental variable should be robustly associated with the exposure. Assumption 2 indicates that the instrumental variable should not be associated with any confounders. Assumption 3 indicates that the instrumental variable used should affect the risk of the outcome only through the risk factor, not through alternative pathways. Regarding causal inference, the MR design resembles that of an RCT; specifically, the random allocation of genetic variants in MR mimics the randomisation process of RCTs, which minimises confounding effects. Source: Manhattan plot reproduced from Ikram et al [75], available under a CC BY 2.5 licence (https://creativecommons.org/licenses/by/2.5/). This figure is available as a downloadable slide

Mendelian randomisation (MR) is an epidemiological method that can strengthen causal inference by using genetic variants as instrumental variables [5]. An instrumental variable is a variable that satisfies three main conditions: (1) it is associated with the exposure (relevance assumption); (2) it does not share a common cause with the outcome (independence assumption); and (3) it is related to the outcome only through the exposure (exclusion restriction assumption) (Fig. 1). The text box summarises the common terms used in MR studies and their key concepts and limitations. As genetic variants are randomly assorted at conception and thus are generally unassociated with environmental and self-adopted factors, MR is believed to be less affected by measured and unmeasured confounding factors. This narrative review aims to summarise the evidence on potential causal risk factors for diabetes by integrating published MR studies on type 1 and 2 diabetes, and to reflect on future perspectives of MR studies on diabetes.

## Causal exposures and risk factors for type 1 diabetes

Because there is a strong genetic component in type 1 diabetes, MR studies of type 1 diabetes are limited and only a few potentially modifiable risk factors have been identified (Table 1). Low birthweight [6], childhood obesity [6, 7] and a higher abundance of the *Bifidobacterium* genus [8] have been associated with an increased risk of type 1 diabetes. MR studies have found no associations of adult body size [6], features of the liver or pancreas [9] and serum 25-hydroxyvitamin D levels [10] with type 1 diabetes.

**Table 1:** MR studies on the causes of type 1 diabetes

Exposure	First author, year of publication	SNPs	Cases/controls	IVW estimate, OR (95% CI)	Robustness in sensitivity analyses^a^
Birthweight	Richardson, 2022 [6]	161	15,573/158,408	0.66 (0.47, 0.92) per SD increase	Yes
Childhood obesity	Censin, 2017 [7]^b^	23	5913/8828	1.32 (1.06, 1.64) per SD increase	Yes
Childhood body size	Richardson, 2022 [6]	280	15,573/158,408	2.05 (1.20, 3.50) per change in body size category (thinner, average, plumper)	Yes
Adult body size	Richardson, 2022 [6]	515	15,573/158,408	1.60 (1.05, 2.45) per change in body size category (thinner, average, plumper)	No
Liver fat	Martin, 2022 [9]^b^	10	9358/15,705	1.06 (0.90, 1.27) per SD increase	Yes
Liver volume	Martin, 2022 [9]^b^	11	9358/15,705	0.92 (0.67, 12.7) per SD increase	Yes
Pancreas fat	Martin, 2022 [9]^b^	9	9358/15,705	1.26 (0.82, 1.93) per SD increase	No
Pancreas volume	Martin, 2022 [9]^b^	16	9358/15,705	1.55 (0.85, 2.85) per SD increase	No
Serum 25OHD levels	Manousaki, 2021 [10]^b^	69	9358/15,705	1.09 (0.86, 1.40) per SD decrease	Yes
Circulating protein biomarkers	Yazdanpanah, 2022 [11]^b^	1 *cis*-pQTL for each	9358/15,705	1.66 (1.36, 2.03) per SD increase in SIRPG; 1.97 (1.48, 2.62) per SD increase in IL27.EBI3; 0.80 (0.73, 0.88) per SD increase in CTRB1	Yes
Gut microbiota	Xu, 2022 [8]^b^	5	6683/12,173 (discovery); 3041/449,223 (replication)	1.61 (1.34, 1.92) for higher abundance of the *Bifidobacterium* genus	Yes
Telomere length	Haycock, 2017 [64]^b^	6	7514/9045	0.71 (0.51, 0.98) per SD increase	Not reported

^a^Robustness in sensitivity analyses was defined as the association remaining stable in any of the following three sensitivity analyses: weighted median, MR-Egger and MR-PRESSO. We defined the sensitivity analyses as robust for studies on metabolites and proteins as the associations remained stable in the analyses based on different data sources

^b^Study was based on summary-level data

25OHD, 25-hydroxyvitamin D; CTRB1, chymotrypsinogen B1; IL27.EBI3, IL-27 Epstein–Barr virus-induced 3; IVW, inverse variance weighted; pQTL, protein quantitative trait loci; SIRPG, signal regulatory protein gamma

A protein-wide MR study examined the associations of 1611 circulating protein biomarkers with the risk of type 1 diabetes and identified associations for signal regulatory protein gamma, IL-27 Epstein–Barr virus-induced 3 and chymotrypsinogen B1 [11]. These findings linking certain viral infections, particularly by enteroviruses (e.g. coxsackievirus), with the risk of type 1 diabetes are consistent with recent observational studies [12], thus providing an avenue to better understand and prevent this disease.

## Causal exposures and risk factors for type 2 diabetes

Most MR studies on glycaemic outcomes have focused on type 2 diabetes. Our previous exposure-wide MR study examined the associations of 97 exposures with risk of type 2 diabetes using data from the DIAbetes Genetics Replication And Meta-analysis (DIAGRAM) consortium (74,124 cases and 824,006 controls). In total, 34 factors that were possibly causally associated with the risk of type 2 diabetes were identified [13]. In Table 2 we summarise and update the associations of a wide range of exposures with type 2 diabetes from MR studies on diabetes.

**Table 2:** MR studies on the causes of type 2 diabetes

Exposure	First author, year of publication	SNPs	Cases/controls	IVW estimate, OR (95% CI)	Robustness in sensitivity analyses^a^
Somatic health status					
Systolic blood pressure	Aikens, 2017 [65]^b^	28	37,293/125,686	1.02 (1.01, 1.03) per 1 mmHg increase	Yes
HDL-cholesterol	Fall, 2015 [14]^b^	140	34,840/114,981 (EUR)	0.86 (0.79, 0.94) per SD increase	Yes
	Soremekun, 2022 [15]^b^	18	23,305/30,140 (AA)	0.92 (0.84, 0.99) per SD increase	Yes
Uric acid levels	Sluijs, 2015 [66]	24	41,508/NA	1.01 (0.87, 1.16) per 1 mg/dl (59.5 μmol/l) increase	Not reported
Rheumatoid arthritis	Zhang, 2022 [67]^b^	70	74,124/824,006	1.04 (1.02, 1.05) per log-odds	Yes
Liver function	De Silva, 2019 [68]	3 for AST	64,094/607,012	1.25 (1.14, 2.38) per SD increase in AST	Yes
		4 for ALT		1.45 (1.10, 1.92) per SD increase in ALT	
		13 for ALP		0.91 (0.86, 0.97) per SD increase in ALP	
		22 for GGT		0.92 (0.80, 1.06) per SD increase in GGT	
Liver volume	Martin, 2022 [9]^b^	11	55,005/400,308	1.27 (1.08, 1.49) per SD increase	Yes
Pancreas volume	Martin, 2022 [9]^b^	17	55,005/400,308	0.76 (0.62, 0.96) per SD increase	Yes
Polycystic ovary syndrome	Zhu, 2021 [69]^b^	14 for EUR	74,124/824,006 (EUR)	0.97 (0.92, 1.01) per log-OR in EUR	Yes
		13 for EA	77,418/356,122 (EA)	0.98 (0.96, 1.01) per log-OR in EA	
Gallstone disease	Wang, 2019 [70]	8	2233/4767 (EA)	1.17 (0.90, 1.52) per SD increase in log-OR	Not reported
Circulating bilirubin levels	Abbasi, 2015 [17]	1	210/3171	0.75 (0.62, 0.92) per SD increase in log-transformed levels	Not reported
Age at menarche	Cao, 2020 [71]^b^	122	26,676/132,532	0.83 (0.78, 0.88) per 1 year increase	Not reported
Testosterone levels	Ruth, 2020 [23]^b^	254 (women)	17,790/243,645 (women)	1.37 (1.22, 1.53) per SD increase in women	Yes
		231 (men)	34,990/150,760 (men)	0.86 (0.76, 0.98) per SD increase in men	
SHBG levels	Yuan, 2022 [24]	450	103,290/1,007,191	0.49 (0.43, 0.57) per nmol/l increase	Yes
Sleep and mental health					
Sleep duration	Yuan, 2020 [13]^b^	73	74,124/824,006	0.83 (0.62, 1.12) per h/night	Yes
Short sleep (<7 h/night)	Yuan, 2020 [13]^b^	25	74,124/824,006	1.14 (0.92, 1.41) per log-OR	Yes
Long sleep (>9 h/night)	Yuan, 2020 [13]^b^	8	74,124/824,006	0.79 (0.47, 1.34) per log-OR	Yes
Insomnia	Yuan, 2020 [13]^b^	208	74,124/824,006	1.17 (1.11, 1.23) per log-OR	Yes
Depression	Tang, 2020 [72]^b^	96	74,124/824,006	1.26 (1.10, 1.43) per log-OR	Yes
Adiposity-related factors					
Birthweight	Huang, 2019 [31]	7	28,806/52,691 (EUR)	1.96 (1.42, 2.71) per SD decrease in EUR	Yes
			12,349/27,317 (EA)	1.39 (1.18, 1.62) per SD decrease in EA	
Childhood obesity	Geng, 2018 [25]^b^	15	34,840/114,981	1.83 (1.46, 2.30) per SD increase	Yes
Adulthood overall obesity (BMI)	Wainberg, 2019 [26]	57	13,982/273,412	1.31 (1.11, 1.53) per kg/m^2^ increase for BMI <25 kg/m^2^	Not reported
				1.36 (1.28, 1.45) per kg/m^2^ increase for BMI 25–30 kg/m^2^	
				1.25 (1.20, 1.31) per kg/m^2^ increase for BMI >30 kg/m^2^	
Adulthood overall obesity (BMI)	Dale, 2017 [27]	97	34,840/114,981	1.98 (1.41, 2.78) per SD increase	Yes
Adulthood central obesity (WHR adjusted for BMI)	Dale, 2017 [27]	49	34,840/114,981	1.82 (1.38, 2.24) per SD increase	Yes
Liver fat	Martin, 2022 [9]^b^	10	55,005/400,308	1.27 (1.08, 1.49) per SD increase	Yes
Pancreas fat	Martin, 2022 [9]^b^	9	55,005/400,308	1.02 (0.76, 1.37) per SD increase	Yes
Whole-body fat mass	Larsson, 2020 [29]^b^	581	26,676/132,532	1.96 (1.54, 2.50) per kg/m^2^ increase	
Visceral fat mass	Karlsson, 2019 [28]	102	6473/101,623 (men)	2.50 (1.98, 3.14) per 1 kg increase for men	Yes
			3044/106,371 (women)	7.34 (4.48, 12.0) per 1 kg increase for women	
Plasma adiponectin	Nielsen, 2021 [30]	10	62,829/596,487	1.26 (1.01, 1.57) per log-transformed decrease	No
Lifestyle factors					
Smoking initiation	Yuan, 2019 [33]^b^	377	74,124/824,006	1.28 (1.20, 1.37) per SD increase in log-OR	Yes
Lifetime smoking index	Yuan, 2020 [13]^b^	126	74,124/824,006	1.61 (1.36, 1.91) per SD increase	Yes
Alcohol consumption	Yuan, 2020 [13]^b^	83	74,124/824,006	1.08 (0.80, 1.45) per SD increase in log drinks/week	No
Coffee consumption	Yuan, 2020 [13]^b^	12	74,124/824,006	1.59 (1.09, 2.32) per 50% increase	Yes
Milk consumption	Vissers 2019 [73]	1	9686/12,134	0.99 (0.93, 1.05) per 15 g/day increase	No sensitivity analysis
Vigorous physical activity	Meisinger, 2020 [32]^b^	8	74,124/824,006	0.83 (0.56, 1.23) per SD increase in accelerations	Yes
Nutritional factors					
Serum 25OHD levels	Yuan, 2019 [37]^b^	7	74,124/824,006	0.94 (0.88, 0.99) per SD increase	Yes
Serum vitamin K levels	Zwakenberg, 2019 [40]	4	69,547/551,336	0.93 (0.89, 0.97) per ln nmol/l increase	Yes
Serum iron levels	Wang, 2021 [41]^b^	3	74,124/824,006	1.07 (1.02, 1.12) per SD increase	Yes
Serum fatty acid levels	Yuan, 2020 [42]^b^	1–4	74,124/824,006	Eight out of ten fatty acids identified: 0.86 (0.81, 0.91) per SD increase in palmitoleic acid	Influenced by *FADS1*/*2* genes
Amino acids	Lotta, 2016 [50]	5 (isoleucine)	47,877/267,694	1.44 (1.26, 1.65) per SD increase in isoleucine	Yes
		1 (leucine)		1.85 (1.41, 2.42) per SD increase in leucine	
		1 (valine)		1.54 (1.28, 1.84) per SD increase in valine	
Amino acids	Yuan, 2020 [13]^b^	1-2	74,124/824,006	0.51 (0.45, 0.58) per SD increase in alanine	No sensitivity analysis
				1.15 (1.03, 1.29) per SD increase in phenylalanine	
				0.86 (0.76, 0.98) per SD increase in tyrosine	
Inflammatory and protein biomarkers					
IGF-1 levels	Larsson, 2020 [46]^b^	416	74,124/824,006	1.14 (1.05, 1.24) per SD increase	Yes
C-reactive protein	Noordam, 2018 [74]^b^	15	12,171/56,862	1.15 (0.93, 1.42) per ln mg/l increase	No
IL-1 receptor antagonist	Yuan, 2020 [13]^b^	2	74,124/824,006	1.13 (1.01, 1.27) per 0.22 SD increase	No sensitivity analysis
IL-6 receptor	Swerdlow, 2012 [49]	1	12,859/86,807	0.97 (0.94, 1.00) per minor allele	No sensitivity analysis
Circulating protein biomarkers	Ghanbari, 2022 [58]^b^	1 for each	74,124/824,006	20 of 1089 proteins identified	Yes

^a^Robustness in sensitivity analyses was defined as the association remaining stable in any of the following three sensitivity analyses: weighted median, MR-Egger and MR-PRESSO. We defined the sensitivity analyses as robust for studies on metabolites and proteins as the associations remained stable in the analyses based on different data sources. No sensitivity analysis means that the sensitivity analysis could not be performed because of a limited number of genetic variants

^b^Study based on summary-level data

25OHD, 25-hydroxyvitamin D; AA, African American; ALP, alkaline phosphatase; ALT, alanine aminotransferase; AST, aspartate aminotransferase; EA, East Asian; EUR, European; GGT, gamma-glutamyl transferase; NA, not available; SHBG, sex hormone-binding globulin

### Somatic and psychological health status

The results of MR studies of somatic and psychological health status in relation to type 2 diabetes are summarised in Table 2. Contradictory associations were reported for LDL-cholesterol and type 2 diabetes, with an inverse association observed in a European population and a positive association in an African population [13–15]. A recent study further identified that the diabetogenic effect of low levels of LDL-cholesterol might be mediated by increased BMI [16]. Lower levels of bilirubin (a marker of liver function) [17], testosterone [18] and thyrotropin [19] were associated with an increased risk of type 2 diabetes in some MR studies, but not all [13, 20–22]. Sex-specific associations were observed for testosterone [23, 24], with an increased risk of type 2 diabetes in women but a decreased risk in men with higher testosterone levels [23]. Insomnia, but no other sleep-related traits, was associated with type 2 diabetes [13].

### Adiposity-related factors

Similar to the large body of evidence from prospective observational studies, childhood obesity, adulthood overall obesity and central obesity, excessive liver fat and whole-body and visceral fat mass were all associated with an increased risk of type 2 diabetes [9, 25–29]. Plasma levels of adiponectin, an adipocyte-secreted hormone, are decreased in individuals with obesity, which was associated with an increased risk of type 2 diabetes [30]. However, this association was inconsistent in MR sensitivity analyses [30], suggesting that the association may be biased by pleiotropy (e.g. from fat mass). Several MR studies have found that lower birthweight, independent of adult body weight, is associated with a higher risk of type 2 diabetes [31], which may suggest a role of the uterine environment and fetal development in the development of type 2 diabetes.

### Lifestyle and nutritional factors

MR studies have strengthened the causal role of cigarette smoking in type 2 diabetes and failed to convincingly confirm the effects of physical activity and alcohol and coffee consumption on type 2 diabetes risk [13, 32, 33]. Although alcohol consumption instrumented by 83 SNPs was not associated with type 2 diabetes, the main SNP that associates with higher alcohol consumption and alcohol abuse in European populations (i.e. rs1229984 in the *ADH1B* gene) was significantly associated with an increased risk of disease [13]. A robust inverse association between coffee consumption and type 2 diabetes risk has been reported in many observational studies [34]. However, genetically predicted higher coffee consumption was not associated with a decreased risk of type 2 diabetes in MR studies [13, 35]. Pleiotropic effects of the SNPs used may cause this lack of association (e.g. from fat mass or other hot beverages or caffeine-containing drinks) and the inverse relationship between genetically proxied coffee consumption and plasma caffeine levels (i.e. the genetic variants with the strongest association with higher coffee consumption are associated with lower plasma caffeine levels) [36].

An MR study found an inverse association between circulating 25-hydroxyvitamin D levels and type 2 diabetes risk [37], and this association might be driven by the vitamin D synthesis pathway [37–39]. Lower levels of vitamin K1 (phylloquinone) [40] and higher levels of iron [41] were associated with an increased risk of type 2 diabetes. Eight out of ten plasma fatty acids were found to be associated with type 2 diabetes; however, the associations, with the exception of palmitoleic acid, were driven by SNPs in the *FADS1*/*2* genes [42]. Thus, whether these associations were biased by this pleiotropic gene, which encodes a key enzyme in fatty acid metabolism, remains unknown [43].

Despite the popularity of MR studies for investigating dietary and lifestyle exposures in diabetes and cardiometabolic diseases, there are unique challenges in such studies of these time-varying, compositional and intercorrelated exposures [44]. For example, MR analyses of nutritional exposures based on genetic instruments for a single measure of diet collected in midlife bear an underlying assumption that, on average, the dietary assessment tool is representative of long-term habitual intake. Furthermore, like many behavioural exposures, nutrition is intercorrelated with numerous other lifestyle and environmental factors. Recent studies have documented that confounding and reverse causation affecting traditional epidemiological studies may also impact genetic associations [45]. A recent study has shown that half of the genetic variants associated with diet are the consequence of increased BMI and that it is possible to use genetics to correct for confounding and reverse causation to strengthen genetic correlations and causal inference [45].

### IGF-1 and inflammatory biomarkers

Genetically predicted elevated levels of IGF-1, a peptide hormone similar in molecular structure to insulin, were positively associated with the risk of type 2 diabetes [46]. Given the heterogeneous effects of IGF-1-associated SNPs on type 2 diabetes, a recent MR analysis examined several clusters of IGF-1-associated SNPs in relation to type 2 diabetes and specified that this overall positive association might be explained by pathways related to amino acid metabolism and genomic integrity [47]. However, the main cluster of IGF-1-associated SNPs that were associated with a decreased risk of type 2 diabetes mapped to the growth hormone signalling pathway [47], possibly mediated by pleiotropic effects from fat mass, as growth hormone secretion is decreased in obesity [48].

As for inflammatory biomarkers, the IL-1 and IL-6 pathways may be involved in the development of type 2 diabetes [13, 49], even though the evidence is weak. One additional minor allele of the *IL6R* SNP rs7529229 (corresponding to the effect of taking tocilizumab 4–8 mg/kg every 4 weeks) was suggestively associated with a reduced risk of type 2 diabetes (OR 0.97, 95% CI 0.94, 1.00), which implied a possible role of IL-6 receptor blockade in type 2 diabetes prevention.

### Circulating metabolites and proteins

One of the first demonstrations of the use of MR to study circulating metabolites was in relation to the previously reported epidemiological association between plasma levels of branched-chain amino acids (BCAAs) and the risk of type 2 diabetes [50]. In an MR analysis using genetic variation at the *PPM1K* locus (which encodes a mitochondrial phosphatase that activates branched-chain α-ketoacid dehydrogenase [BCKD]), an increase in leucine, isoleucine and valine levels was associated with an increased odds of type 2 diabetes [50]. However, given that BCKD has a range of substrates besides leucine, isoleucine and valine, untangling which of these substrates causes type 2 diabetes is challenging. A separate MR analysis of BCAAs showed that higher BCAA levels have no causal effects on insulin resistance but, rather, genetically raised insulin resistance drives higher circulating fasting BCAA levels [51]. A metabolome-wide MR approach confirmed evidence of the strong reverse causal effect, indicating that the genetic predisposition to type 2 diabetes may trigger early changes in valine and leucine [52]. Other products of amino acid catabolism, such as 2-aminoadipic acid (2-AAA) or α-hydroxybutyrate, are strongly associated with incident type 2 diabetes in observational studies [53], but MR studies have failed to demonstrate evidence of causality [54]. There are many reasons for the discrepancies between observational studies and MR studies, but the fact that observational studies have been conducted in a mixture of individuals with normoglycaemia and impaired glucose tolerance could explain these differences. A study in the Framingham cohort restricted to individuals with strict normoglycaemia at baseline (fasting glucose <5.6 mmol/l) provided evidence of a subset of 19 metabolites associated with the risk of diabetes among apparently healthy individuals [55]. Pathway enrichment analyses and MR showed that metabolites in the nitrogen metabolism pathway are causally related to the development of diabetes [55].

Integration of genomic and small molecule data across platforms enables the discovery of regulators of human metabolism and translation into clinical insights. A recent genome-wide meta-analysis of 174 metabolite levels across six cohorts, including up to 86,507 participants, identified ~500 genetic loci influencing metabolite levels [56]. Among many relevant findings for dysglycaemia, the study provided evidence that a missense p.Asp470Asn (rs17681684) variant in the *GLP2R* gene, which encodes the receptor for glucagon-like peptide 2, was associated with a 4% higher type 2 diabetes risk. Findings from a metabolome-wide MR analysis further identified new metabolites that potentially play a causal role in type 2 diabetes, including betaine, glutamic acid, lysine, alanine and mannose [52].

High-throughput detection and quantification of serum proteins in a large human population can provide insight into the molecular processes underlying diabetes risk. A protein-wide MR study examined the associations of 164 proteins with genome-wide association summary statistics available from the independent INTERVAL study and identified 16 proteins as potentially having a causal effect on the development of type 2 diabetes [57]. A recent protein-wide MR study examined the associations of 1089 circulating protein biomarkers with the risk of type 2 diabetes [58]. The analyses identified 20 proteins that might be causally associated with type 2 diabetes. These findings may provide evidence to support therapeutic development in type 2 diabetes.

MR studies on circulating metabolites and proteins usually employ a *cis*-variant located in an encoding gene region as the instrumental variable, which satisfies three key assumptions of MR. However, these MR associations can still be influenced by the genome-wide associations analyses on metabolites and proteins as well as corresponding profiling process (possible bias caused by batch effects) [59] and different high-throughput platforms [60]. Of note, using *cis*-variants as instrumental variables may not always completely rule out horizontal pleiotropy, especially when one gene regulates several metabolites and proteins that are not in a common pathway. In this case, multivariable MR analysis or removing the pleiotropic SNPs may help reduce this bias.

### Gut microbiota and related metabolites

With increasing evidence suggesting that the human gut microbiome plays a role in immune function and metabolic disease, there is a need to discriminate between microbiome features that are causal for disease and those that are a consequence of disease or its treatment. A study including genome-wide genetic data, gut metagenomic sequencing and measurements of faecal short-chain fatty acids showed that a host genetic-driven increase in gut production of butyrate was associated with improved insulin response following an oral glucose test. In contrast, abnormalities in the production or absorption of propionate were causally related to an increased risk of type 2 diabetes [61]. Another two-sample MR study identified seven genera of gut microbiota nominally associated with type 2 diabetes [62]. For gut microbiota-related metabolites, a separate study found that genetically predicted higher trimethylamine *N*-oxide and carnitine levels were not associated with higher odds of type 2 diabetes. However, the study found possible associations of high choline and low betaine levels with an increased risk of type 2 diabetes [63]. Of note, although many genome-wide association analyses of the gut microbiome have been carried out, high-quality MR studies on the gut microbiome in relation to diabetes are limited [8]. This may raise doubt over the applicability of host genetic variants as an instrumental variable to mimic the function of the gut microbiome.

## Assessment of included MR studies on diabetes

The overall quality of the MR studies included was satisfactory, with careful genetic instrument selection criteria, comparatively large sample sizes and different approaches to testing the robustness of the findings. As for the examination of the assumptions of MR, assumption 1 was usually found to be satisfied by using genetic variants associated with the exposure of interest at the genome-wide significance level. However, there was no unified threshold for linkage disequilibrium of SNPs. Using a high or low threshold of linkage disequilibrium could lead to an inflated rate of type 1 and 2 errors, respectively. As MR analysis can minimise confounding, the associations are less likely to be biased by confounding but cannot be completely immune to this bias, especially when genetic instruments have large pleiotropy effects. Except for studies using individual-level data, whether genetic instruments were primarily associated with other phenotypes or were associated with confounders was rarely examined in these MR studies. The most common bias in MR analysis is horizontal pleiotropy caused by violation of assumption 3, the exclusion restriction assumption, which means that genetic variants affect the outcome through alternative pathways, not only through the exposure of interest. The associations with type 1 and 2 diabetes summarised in this review were robust in sensitivity analyses, and most studies used MR-Egger or MR pleiotropy residual sum and outlier (MR-PRESSO) to detect potential horizontal pleiotropy. Of note, even though statistical methods can detect and minimise the influence of horizontal pleiotropy, instrumental variable selection is a crucial process for reducing the bias. Using genetic variants in genes with well-understood biological functions as instrumental variables usually satisfies the assumptions of MR analysis and thus generates precise and correct associations. However, it is difficult to identify specific genetic variants for certain exposures, especially for health behaviours and complex phenotypes. Therefore, a thoughtful examination of pleiotropy should be conducted in analyses using multiple genetic instruments. Evidence from observational studies and clinical trials should be used in interpreting MR findings. Robust MR findings, in turn, should be examined in clinical trials. In addition, it is tricky to interpret MR results, especially for binary exposures. Given that the exposure in MR analysis is not an exact phenotype but is proxied by the effects of genetic variants on a certain trait, this genetically proxied exposure usually mimics a lifetime chronic effect, which hinders the exploration of time-specific associations.

## Future perspectives

- The null findings in previous MR studies may have been caused by inadequate power, particularly for weak associations of exposures proxied by a few SNPs that explained a small phenotypic variance. For exposures with robust associations in traditional observational studies, the neutral associations in MR studies deserve to be re-examined in well-powered studies with robust genetic instruments for the exposures and large sample sizes for the diabetes outcomes.
- Most previous MR studies were based on summary-level data, which do not allow the exploration of potential non-linear associations (e.g. J- or U-shaped); rather, it can only be assumed that the association is linear without a threshold effect. MR analysis using individual-level data from large-scale biobanks and studies is needed to examine the non-linearity of the associations.
- More effort should be put into MR studies on non-heritable exposures or exposures without genetic association information. For example, MR analyses of the association of diet and physical activity with diabetes risk are warranted.
- Most MR studies have been based on data from European populations. With more and more data available from other populations, such as Asian and African populations, future MR studies are encouraged to include data from multi-ancestry cohorts.
- Even though the associations between protein biomarkers and diabetes risk were examined in a few MR studies [11, 58], more independent verification is needed to confirm these findings. In addition, the intermediate roles of blood proteins and metabolites in the pathways from environmental exposure to diabetes should be investigated to provide evidence for treatment and intervention.
- Even though many statistical approaches, such as the weighted median, MR-Egger, MR-PRESSO, MR-Cluster and contamination mixture methods, have been developed to detect pleiotropy and verify the association with different assumptions, more efforts are needed to generate new statistical approaches to handle pleiotropy and other limitations.

## Conclusion

This review has integrated data from published MR studies on type 1 and 2 diabetes to highlight the many possible causal risk factors for dysglycaemia. While few studies have been conducted for type 1 diabetes, most MR analyses support that social, demographic, metabolic and lifestyle factors are causally associated with the development of type 2 diabetes. More MR studies in multi-ancestry cohorts are needed to examine the role of diet in the development of diabetes. MR investigations based on data on metabolites, protein biomarkers and the gut microbiome may help to illustrate the pathological molecular basis of diabetes.

## Supplementary Information

Figure slide (PPTX 151 kb)

## Abbreviations

BCAA: Branched-chain amino acid
BCKD: Branched-chain α-ketoacid dehydrogenase
MR: Mendelian randomisation
