# Supplementary material for: Causal factors underlying diabetes risk informed by Mendelian randomisation analysis: evidence, opportunities and challenges
Source: Diabetologia. 2023 Feb 14;66(5):800–12. doi: 10.1007/s00125-023-05879-7 (PMC10036461; doi:10.1007/s00125-023-05879-7)
Supplement: Supplementary file 1 — (PPTX 151 kb) [file 125_2023_5879_MOESM1_ESM.pptx]

## Slide 1
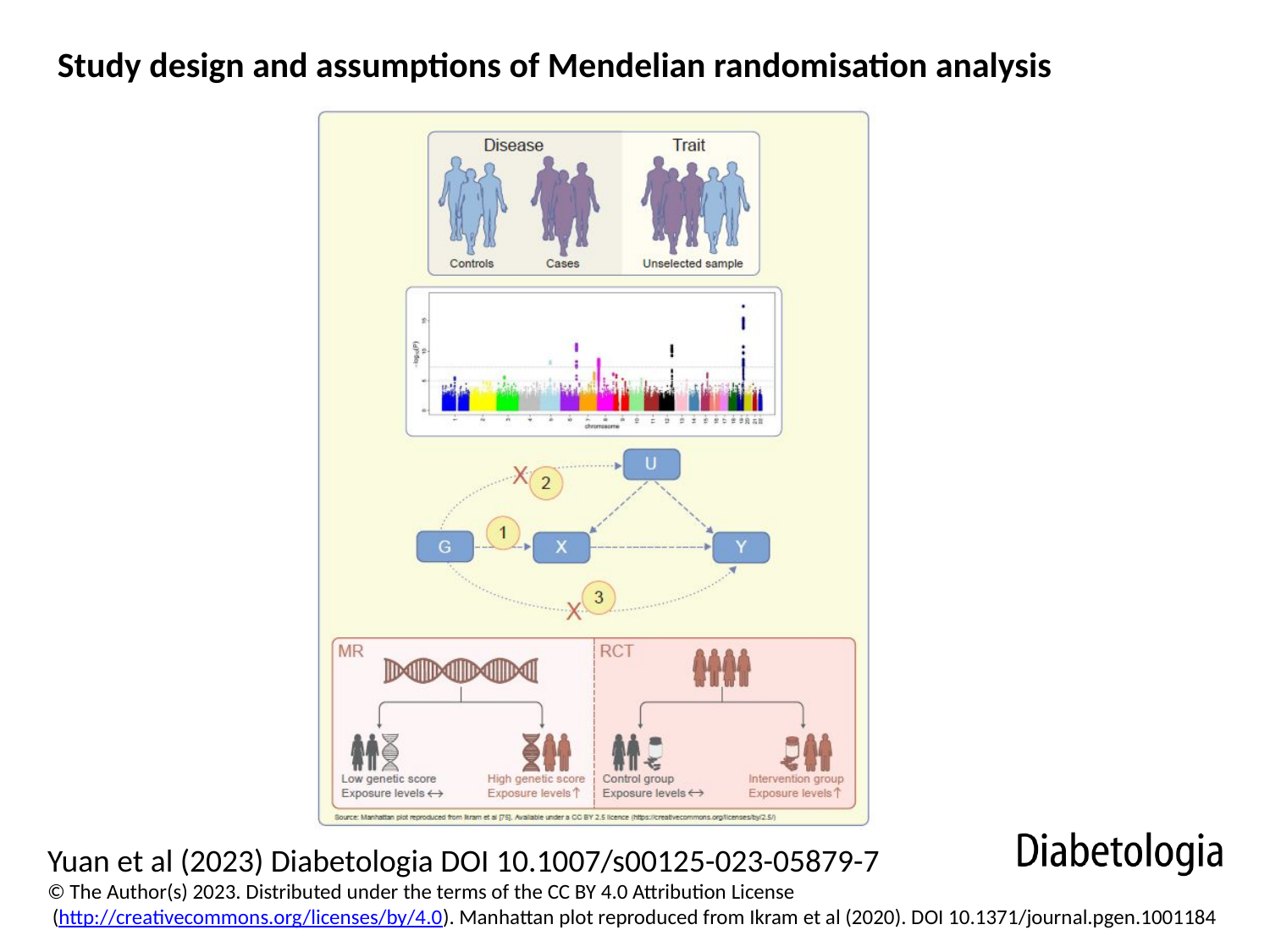

Study design and assumptions of Mendelian randomisation analysis
Yuan et al (2023) Diabetologia DOI 10.1007/s00125-023-05879-7
© The Author(s) 2023. Distributed under the terms of the CC BY 4.0 Attribution License
 (http://creativecommons.org/licenses/by/4.0). Manhattan plot reproduced from Ikram et al (2020). DOI 10.1371/journal.pgen.1001184
